# Dietary Fructooligosaccharide Is Associated with Improved Insulin Resistance, Reduced *Romboutsia ilealis* Abundance, and Modulation of GLP-1 Production in High-Fat Diet-Induced Obese Mice

**DOI:** 10.3390/nu18193258

**Published:** 2026-10-02

**Authors:** Lais Yuki Tuzino Kamia, Lauren Lewis, Zack Nobles, Gar Yee Koh

**Affiliations:** Nutrition and Foods Program, School of Family and Consumer Sciences, College of Applied Arts, Texas State University, San Marcos, TX 78666, USA; guw13@txstate.edu (L.Y.T.K.); hev30@txstate.edu (L.L.); j_n292@txstate.edu (Z.N.)

**Keywords:** fructooligosaccharides, glucagon-like peptide-1 (GLP-1), insulin resistance, *Romboutsia ilealis*, diet-induced obesity, gut microbiota

## Abstract

Background/Objectives: Obesity disrupts glucose homeostasis and insulin signaling, partly through altered gut hormone secretion and intestinal inflammation. Our previous work demonstrated that fructooligosaccharides (FOS), a prebiotic fiber, suppressed colonic inflammation, and modulated gut microbiota when administered with vitamin D supplementation. However, the effects of FOS on metabolic alterations, glucagon-like peptide-1 (GLP-1) production under obesogenic stimulation remains unclear. Here, we examined the effects of FOS on insulin resistance and GLP-1 production in high-fat diet (HFD)—induced obese mice. Methods: Six-week-old male and female C57BL/6J mice were assigned to control or HFDs ± 5% FOS for 10 weeks. Body weight, visceral fat, fasting glucose, insulin, triglycerides, and insulin resistance index (HOMA-IR) were assessed. Serum GLP-1 was quantified by ELISA. Colonic expressions of *Glp1r*, *Pi3k*, and *Akt* were evaluated by qRT-PCR and western blotting, respectively. Cecal abundance of *Akkermansia muciniphila* (AM), *Dubosiella newyorkensis* (DN), and *Romboutsia ilealis* (RI) was quantified by qPCR. Results: FOS attenuated HFD-induced metabolic dysfunction, lowering serum insulin and HOMA-IR in both sexes. Notably, FOS elicited sex-dependent physical improvements of which it suppressed body weight gain by 10% in male mice and reduced visceral fat accumulation by 36% in female mice. The phenotypic changes in male mice were associated with an increase in GLP-1. Further, FOS modulated colonic PI3K/Akt signaling in a sex-dependent manner and downregulated HFD-associated increases in *Glp1r* mRNA expression. Consistent with our previous work, FOS suppressed RI abundance, and this reduction was associated with lower colonic *Tlr4* mRNA expression and improved insulin-resistance index. Conclusions: FOS improves insulin sensitivity in HFD-induced obesity and specifically enhanced GLP-1 production in male mice. Though the mechanism remained elucidated, the observed phenotype changes may be attributed to a combination of altered colonic PI3K/Akt pathways and targeted bacterial shifts. These effects appear to be sex-dependent and support FOS as a promising dietary strategy for maintenance of metabolic health.

## 1. Introduction

Obesity has become one of the most pressing metabolic health challenges, affecting over 1 billion people worldwide, and remains a major driver of insulin resistance, dyslipidemia, type 2 diabetes, and related chronic disorders [1,2]. In the United States, the prevalence of obesity continues to rise, with more than 42% of adults currently classified as obese (body mass index [BMI] > 30 kg/m^2^) [3]. Although obesity is often described as excess body weight, its biological impact extends well beyond adiposity alone. In the obese state, adipose tissue undergoes expansion and functional remodeling that promote chronic low-grade inflammation, alters lipid handling, and impairs glucose homeostasis [4,5]. A central feature of obesity-associated metabolic dysfunction is impaired insulin signaling [6]. As adipocytes enlarge and become stressed, they release inflammatory mediators and free fatty acids that interfere with insulin signaling in adipose tissue, liver, and skeletal muscle [2,6]. A commonly affected pathway is the phosphoinositide 3-kinase/protein kinase B (PI3K/Akt) pathway, which normally promotes glucose uptake, supports lipid homeostasis, and mediates key downstream actions of insulin [7]. In obesity, inflammatory signaling disrupts this pathway and contributes to hyperinsulinemia, impaired glucose disposal, increased triglycerides, and abnormal fat accumulation [6,8]. This is especially relevant for visceral adiposity, which is more strongly associated with metabolic complications and inflammatory burden than fat stored in less metabolically active depots [4,9]. Consequently, interventions that reduce visceral fat while improving insulin signaling may be particularly valuable for limiting obesity-related disease progression [7].

Glucagon-like peptide-1 (GLP-1) has attracted intense attention because it sits at the intersection of intestinal physiology, insulin regulation, and energy metabolism [10,11]. GLP-1 is secreted by enteroendocrine L-cells and is best known for enhancing glucose-dependent insulin secretion, but its actions extend beyond the pancreas to appetite regulation, lipid metabolism, and insulin sensitivity [12,13]. Experimental work links GLP-1 signaling to activation of PI3K/Akt and restoration of downstream metabolic responses in insulin-sensitive tissues [11,14]. This makes GLP-1 especially interesting in obesity, where the usual insulin-signaling machinery is already compromised by inflammation and lipotoxic stress [10]. Pharmacologic therapies with GLP-1 receptor agonists have shown successful benefits for weight loss and glycemic control [15,16,17,18]. However, the expense, long-term use, and limited accessibility strengthened interest in dietary strategies that target endogenous GLP-1 signaling through physiologic routes [19,20,21]. One possible route for stimulating endogenous GLP-1 secretion and restoring host metabolic signaling involves the gut microbiota [10,12,14]. In healthy individuals, gut microbiota maintains a diverse, balanced community, which promotes metabolic health through production of beneficial metabolites and maintenance of intestinal barrier integrity [20,22]. Under obesogenic conditions, high-fat feeding can shift microbial composition towards dysbiosis, compromising intestinal barrier integrity, thereby favoring translocation of pro-inflammatory bacterial products and amplifying metabolic dysfunction [23,24]. This process is important because it provides a plausible explanation for how a dietary pattern can alter host metabolism not only through calories, but also through intestinal signaling pathways that shape insulin resistance and inflammation [23,25,26]. It also raises a mechanistic question that is highly relevant to the present study on whether modifying the gut environment can influence endogenous GLP-1 and, in turn, downstream pathways related to insulin sensitivity and adiposity [13,27].

Defined as non-digestible dietary compounds selectively fermented by beneficial gut microbiota, prebiotics have emerged as a promising therapeutic approach for mitigating obesity and its associated metabolic disorders [28,29]. Fructooligosaccharides (FOS) are short-chain prebiotic carbohydrates that are well known for modulating gut microbial composition and have been associated with beneficial effects on inflammation and metabolic health [19,30]. Prior work identifies FOS as a dietary intervention capable of targeting both the gut microbiota and host metabolic signaling, leading to short chain fatty acids (SCFA) production and establishing a favorable metabolic environment for beneficial microbiota [30,31,32]. One of the central mechanisms by which FOS exerts metabolic benefits is through activation of G-protein-coupled receptor 41 and 43 on intestinal L-cells by SCFA, promoting the release of GLP-1 to promote glucose homeostasis, and satiety [19,33,34,35,36]. Recent work from our laboratory demonstrated that supplementation with 5% FOS upregulated colonic vitamin D receptor expression in HFD-induced obese mice, which is associated with reduced colonic inflammation characterized by the downregulation of TLR4, an innate immune receptor [37,38]. Among the taxa of interest, *Akkermansia muciniphila* (AM) is the most established in obesity literature and is often linked to improved barrier function and metabolic health [39,40,41,42,43]. *Dubosiella newyorkensis* (DN) has emerged as a taxon with context-dependent links to inflammation and host metabolism, and *Romboutsia ilealis* (RI) a diet-responsive bacterium potentially relevant to intestinal metabolic signaling [44,45,46,47]. RI is traditionally categorized as an opportunistic pathobiont that expands during high-fat diet (HFD) induced dysbiosis and correlates with mucosal inflammation [46]. However, more recent evidence indicates that it can also generate bioactive metabolites, activating local receptors to alter metabolic transcripts [47]. Importantly, a previous study from our laboratory by Hanson et al. demonstrated that dietary FOS supplementation in combination with vitamin D suppressed the abundance of RI in healthy mice, an intervention that was also associated with marked alterations in intestinal tight junction signaling, including a ~20-fold increase in ileal occludin mRNA expression, suggesting a potential role in intestinal barrier regulation [38]. Despite these findings, the behavior of RI under concurrent obesogenic challenges, its responsiveness to isolated prebiotic intervention, and its relationship with colonic tissue signaling remain not fully understood. Rather than considering microbiota solely as a broad ecological shift, focusing on these selected organisms may help identify more specific microbial signatures associated with metabolic improvement and intestinal barrier regulation [26,48]. The present study investigated the effects of dietary FOS supplementation on HFD-induced metabolic dysfunction by examining metabolic phenotypes, circulating GLP-1 level, colonic GLP-1 receptor and PI3K/Akt signaling, and the abundance of selected gut bacteria. By focusing on the colon, we sought to determine whether a gut-targeted prebiotic intervention is associated with coordinated changes in the intestinal microbial environment and gut-derived endocrine and intracellular signaling pathways that may contribute to metabolic improvement. Addressing these relationships is essential for developing targeted, mechanistically informed prebiotic strategies to mitigate obesity-related metabolic disease. Here, we hypothesized that dietary FOS supplementation would ameliorate HFD-induced metabolic dysfunction by modulating the GLP-1 signaling axis and PI3K/Akt pathway activity and remodeling key gut bacterial taxa involved in metabolic and inflammatory regulation.

## 2. Materials and Methods

### 2.1. Study Design

Five-week-old male and female C57BL/6J mice (*n* = 10/group/sex) were obtained from The Jackson Laboratory (Bar Harbor, ME, USA) and transported to the Texas State University Comparative Research Facility. Upon arrival, mice were individually housed in polycarbonate cages under controlled environmental conditions: 12-h light/dark cycle, ambient temperature maintained at 22–25 °C, and relative humidity of 40–60%. Individual housing was employed to enable accurate measurement of individual food intake and to prevent coprophagy, which could confound microbiota analyses and metabolic outcomes. Mice were allowed to acclimate to the housing environment for one week prior to randomization and initiation of dietary interventions. At 6-weeks of age, all mice were provided with ad libitum access to water and their assigned experimental diet throughout the study duration. A mouse was found deceased in cage prior to dietary intervention assignment and hence, it was excluded from all analyses. All experimental procedures were approved by the Texas State University Institutional Animal Care and Use Committee (IACUC protocol #9201) and conducted in accordance with the National Institutes of Health Guide for the Care and Use of Laboratory Animals (8th edition).

Samples were collected at the conclusion of the 10-week dietary intervention (age 16 weeks). All mice were subjected to overnight fasting (16 h, 1700 h to 0900 h) with continued access to water to standardize metabolic state prior to tissue collection. Mice were anesthetized with 3–5% isoflurane (Piramal Pharma Solutions, Bethlehem, PA, USA) delivered via precision vaporizer (E-Z Systems, Palmer, PA, USA) until loss of pedal withdrawal reflex was confirmed. Blood was collected via cardiac puncture into a microcentrifuge tube using a 25-gauge needle. Blood samples were allowed to clot at room temperature for 30 min, then centrifuged at 1200× *g* for 15 min at 4 °C to obtain serum. Serum was aliquoted into microcentrifuge tubes and stored at −80 °C until analysis. Following blood collection, mice were euthanized by cervical dislocation under deep anesthesia. The abdominal cavity was immediately opened via midline laparotomy, and visceral adipose tissue depots (perigonadal) were carefully excised, rinsed briefly in ice-cold phosphate-buffered saline (PBS, pH 7.4) to remove blood contamination, blotted dry on sterile gauze, and weighed to the nearest 0.001 g using an analytical balance (Mettler Toledo, Columbus, OH, USA). The distal colon was opened longitudinally using surgical scissors, and the mucosal layer was gently scraped using a glass microscope slide to collect colonic epithelial cells and associated mucosa while minimizing contamination with smooth muscle. Scraped colonic mucosa was immediately snap-frozen in liquid nitrogen and stored at −80 °C for subsequent protein and DNA extraction. Additionally, cecal contents (approximately 100 mg) were collected aseptically, transferred to cryovials, snap-frozen in liquid nitrogen, and stored at −80 °C for DNA extraction and bacterial quantification analysis.

### 2.2. Experimental Diets

The experimental design utilized a 4 × 2 factorial arrangement with four diet experimental groups: (1) control diet (CON), (2) control diet supplemented with 5% FOS (CON-FOS), (3) high-fat diet (HFD), and (4) high-fat diet supplemented with 5% FOS (HFD-FOS), and two sexes (male and female). All experimental diets were custom-formulated and manufactured by Research Diets, Inc. (New Brunswick, NJ, USA; Supplementary Table S1). All diets were γ-irradiated prior to shipment to ensure microbial sterility. Fructooligosaccharides (FOS; Orafti^®^P95, ≥95% oligofructose derived from chicory root, average degree of polymerization 4–8) were supplied by BENEO-Orafti (Tienen, Belgium) to Research Diets for incorporation into experimental diets. The control diet (CON) was based on the AIN-93G purified rodent diet formulation, which provides 16% of total kilocalories from fat (primarily soybean oil), 64% from carbohydrate (primarily cornstarch and maltodextrin), and 20% from protein (casein) (Research Diets catalog #D10012G). The HFD was based on the widely used diet-induced obesity formulation D12492, which provides 60% of total kilocalories from fat (primarily lard and soybean oil), 20% from carbohydrate (primarily maltodextrin and sucrose), and 20% from protein (casein). FOS supplementation was incorporated into both control and HFD matrices at 5% w/w (50 g FOS per kg of diet), which was substituted isocalorically for cornstarch (in CON-FOS) or maltodextrin (in HFD-FOS) to maintain consistent macronutrient profiles and energy density in both diets. The 5% FOS dose was selected based on previous studies from our laboratory demonstrating that this dose: (1) modulates specific gut bacterial populations including *Akkermansia muciniphila* and *Bifidobacterium* species [37,38,49,50], (2) attenuates obesity-induced inflammation and improves metabolic parameters [19,30], and (3) is well-tolerated with minimal gastrointestinal side effects in rodents [30]. Diets were stored at −20 °C until use and provided fresh to mice three times per week to minimize oxidation of dietary lipids and degradation of heat-labile vitamins.

### 2.3. Blood Glucose and Serum Insulin

Blood glucose levels were measured via a blood glucometer (ReliOn Premier Conpact, Walmart Apollo LLC, Bentonville, AR, USA). Blood samples from the cardiac puncture were utilized by applying one drop of blood onto the glucose meter. Levels were viewed and recorded for later analysis of trends.

Serum Insulin was measured using a commercial Ultra-sensitive Mouse Insulin ELISA Kit (Catalog #90080, Crystal Chem, Elk Grove Village, IL, USA). Whole blood was used and centrifuged at 2000× *g* for 10 min to separate the serum. The ELISA assay kit was performed according to the manufacturer’s instructions. The plate was read with a plate reader (BioTek Cytation5, Agilent Technologies Inc, Santa Clara, CA, USA) at 450 nm. To calculate the Homeostatic Model Assessment for Insulin Resistance (HOMA-IR), fasting glucose values were mathematically converted from mmol/L to mg/dL, and fasting insulin values were converted from ng/mL to µU/mL prior to calculation. HOMA-IR was then calculated using the standard formula: HOMA-IR = [fasting glucose (mg/dL) × fasting insulin (µU/mL)]/405.

### 2.4. Quantification of Serum Triglyceride

Serum triglyceride levels were quantified using a commercial Triglyceride Colorimetric Assay Kit (catalog #10010303, Cayman Chemical, Ann Arbor, MI, USA). According to the manufacturer’s instructions, the colorimetric assay was performed using serum samples collected from each mouse. To initiate the reaction, 150 µL of diluted enzyme mixture solution was added to each well. The plates were carefully shaken and incubated for 15 min at room temperature. To collect the data, absorbance was measured at 530–550 nm using a plate reader. A triglyceride standard curve was generated by performing serial dilutions of the triglyceride standard solution provided in the kit, strictly adhering to the manufacturer’s protocol. Corrected absorbance for each sample was determined by subtracting the absorbance of the blank (zero standard) well from the sample’s raw absorbance. Triglyceride concentrations were then calculated using the linear regression equation generated from the standard curve as follows: triglycerides (mg/dL) = [(corrected absorbance) − (y-intercept)]/slope.

### 2.5. Quantification of Circulating GLP-1

To assess total circulation of GLP-1, fasting serum GLP-1 concentrations were quantified using a commercially available mouse GLP-1 ELISA kit (Crystal Chem #81508 Elk Grove Village, IL, USA). According to the manufacturer’s protocol, serum samples were thawed on ice, vortexed gently, and assayed undiluted in duplicate (30 μL per well). Samples and standards were incubated with anti-GLP-1 antibody for 18 h at room temperature with gentle shaking (100 rpm), followed by washing steps and incubation with enzyme-conjugated detection antibody. After substrate addition and color development, absorbance was measured at 450 nm (with 630 nm reference wavelength) using a microplate reader (Cytation 5 Cell Imaging Multi-Mode Reader, BioTek Instruments, Winooski, VT, USA). GLP-1 concentrations were interpolated from the standard curve using a four-parameter logistic regression model (SigmaPlot software version 14.5, Inpixon, Palo Alto, CA, USA).

### 2.6. Real-Time Polymerase Chain Reaction (RT-PCR)

Analysis of gene expression via RT-PCR was performed to examine the effect of FOS supplementation on metabolic signaling pathway as described previously [37,38]. RNA was extracted from lysed colonic tissues and quantified using a Nanodrop Lite Spectrophotometer (Thermo Fisher Scientific, Waltham, MA, USA). Nucleic acid purity was confirmed with an A260/280 absorbance ratio. cDNA was synthesized using a SuperScript IV cDNA Reverse Transcriptase kit (Invitrogen. Waltham, MA, USA). Real-time PCR reactions were performed in duplicate via PowerUp SYBR Detection Reagent (Applied Biosystems, Waltham, MA, USA) at 100 ng per well with QuantStudio^TM^ 3 Real-Time PCR system (Thermo Fisher Scientific, Waltham, MA, USA) to detect the expression of the *Glp1r*, *Pi3k*, and *Akt*, critical mediators of insulin signaling pathways, as well as *Tlr4,* a pro-inflammatory regulator. The thermal cycling condition consisted of an initial denaturation step at 95 °C for 10 min, followed by 40 cycles of denaturation at 95 °C for 15 s, annealing at 60 °C for 1 min, and extension at 72 °C for 30 s. Amplification specificity for all target genes was validated through melt-curve analysis. The specific primer sets for each target are presented in Table 1.

### 2.7. Western Blot

Analysis of protein expression was conducted via Western Blot. Briefly, colonic mucosa was homogenized in 1X RIPA buffer (Thermo Fisher Scientific, Waltham, MA, USA) supplemented with protease and phosphatase inhibitors (Thermo Fisher Scientific, Waltham, MA, USA) and total protein was extracted. Protein quantification was obtained using the BCA assay, following the manufacturer’s instructions (Thermo Fisher Scientific, Waltham, MA, USA). Equal amounts of protein lysate (45 µg per lane) were loaded on a 4–20% tris-glycine gradient sodium dodecyl sulfate–polyacrylamide gel and transferred onto a nitrocellulose membrane (Invitrogen, Waltham, MA, USA). Membranes were probed with primary pAkt (ser473; Cat#9271, Cell Signaling Technology, Boston, MA, USA), total Akt (Cat#9272, Cell Signaling Technology, Boston, MA, USA), PI3K (p85; Cat#4257 Cell Signaling Technology, Boston, MA, USA), GLP-1R (Cat#PA5-97112, Invitrogen, Rockford, IL, USA) and GAPDH (Cat#SC-32233, Santa Cruz Biotechnology, Dallas, TX, USA) antibodies. All primary antibodies were rabbit-derived, except GAPDH was mouse-derived. Subsequently, membrane was incubated with anti-rabbit (Cat#32460, Invitrogen, Rockford, IL, USA) or anti-mouse (Cat#NXA931V, Cytiva, Marlborough, MA, USA) secondary antibodies, respectively for 60–90 min. All antibodies were diluted to 1:1000, unless otherwise specified. Proteins were visualized with enhanced chemiluminescence reagents West Pico Plus or West Femto (Thermo Fisher Scientific, Waltham, MA, USA) using a Gel-Doc Image Analysis System (Fotodyne Incorporated, Hartland, WI, USA). Densitometry of protein bands was quantified using ImageJ software (version 1.53k, National Institutes of Health, Bethesda, MD, USA). Data were representative of two independent experiments, and the same membrane were probed for multiple target proteins. Target protein expression was normalized to the independent loading control, GAPDH, and expressed as a fold-change relative to the control (CON) group. To determine Akt activation, phosphorylated Akt (pAkt) was normalized directly to total Akt to yield a pAkt/total Akt ratio, representing the proportion of Akt in the active, phosphorylated state.

### 2.8. Cecal DNA Extraction and PCR Analysis

Bacterial genomic DNA was extracted from frozen cecal content samples using the QIAamp DNA Stool Mini Kit (QIAGEN, Hilden, Germany; catalog #51504) according to the manufacturer’s instructions, which are optimized for efficient lysis of bacterial cells and removal of PCR inhibitors present in intestinal digesta. Purified bacterial DNA was stored at −20 °C until quantification of specific bacterial taxa by quantitative PCR (qPCR). DNA concentration and purity were determined using a NanoDrop Lite spectrophotometer (Thermo Fisher Scientific, Waltham, MA, USA). Quantitative PCR was performed on a QuantStudio™ 3 Real-Time PCR System (Thermo Fisher Scientific) using PowerUp™ SYBR™ Green Master Mix (Thermo Fisher Scientific, catalog #A25742). The relative abundance of each target bacteria was normalized to universal Eubacteria abundance. The primers used for bacteria analysis are shown in Table 2 and were previously validated in our laboratory [38].

### 2.9. Statistical Analysis

All statistical analyses were performed using SigmaPlot software version 14.5 (Inpixon, Palo Alto, CA, USA). Prior to statistical testing, all data were assessed for normality using the Shapiro-Wilk test and for equality of variances using the Brown-Forsythe test. Data that did not meet normality assumptions, specifically serum insulin and HOMA-IR, were log_10_-transformed prior to analysis to achieve normality. All data were evaluated via two-way analysis of variance (ANOVA) with two independent factors: (1) Diet (four levels: CON, FOS + CON, HFD, FOS + HFD) and (2) Sex (two levels: male, female). Statistical significance for main effects and interactions was defined as α = 0.05. Tukey’s Honestly Significant Difference (HSD) post hoc test was performed to identify pairwise differences. Body weight changes were analyzed via two-way repeated measured ANOVA (diet · week) for each sex. For quantitative real-time PCR (qRT-PCR) data, statistical analyses were conducted using ΔCt values calculated as ΔCt = Ct (target gene) − Ct (*Gapdh*) prior to fold-change calculation for each target gene (*Glp1r*, *Pi3k*, *Akt*). Results are reported as mean ± standard error of the mean (SEM) fold change relative to the control male group (CON males = 1.0 baseline reference), calculated using the ΔΔCt method. Due to limited tissues and samples availability, randomly selected subset at *n* = 5 per group per sex were utilized for analyses of GLP-1, gene expressions, and bacteria abundance, and *n* = 4 per diet per sex for western blot analyses.

To explore potential relationships between gut microbiota composition and host metabolic signaling, Pearson’s correlation coefficients were calculated across individual mice with available paired observations for each pair of variables between cecal bacterial abundance (relative abundance of AM, DN, and RI) and (1) fasting serum GLP-1 concentrations, (2) colonic gene expression of *Glp*-*1r*, *Pi3k*, *Akt*, and *Tlr4* using 2^−ΔΔCt^ values, and (3) colonic protein expression metrics, including the pAkt/total Akt ratio and PI3K and GLP-1R protein levels normalized to GAPDH. The relative mRNA expression of colonic *Tlr4* used in this study was included in Supplemental Table S2. For correlation analyses, sample size may vary depending on the availability of data for each measured variables ranging from 26–42 paired observations. All data are presented as mean ± SEM unless otherwise indicated. Data points deviating by more than two standard deviations from the mean were excluded from statistical analyses as outliers.

## 3. Results

### 3.1. FOS Suppressed HFD-Induced Body Weight Gain and Elevation of Serum Triglycerides in Male Mice

**Figure 1:**
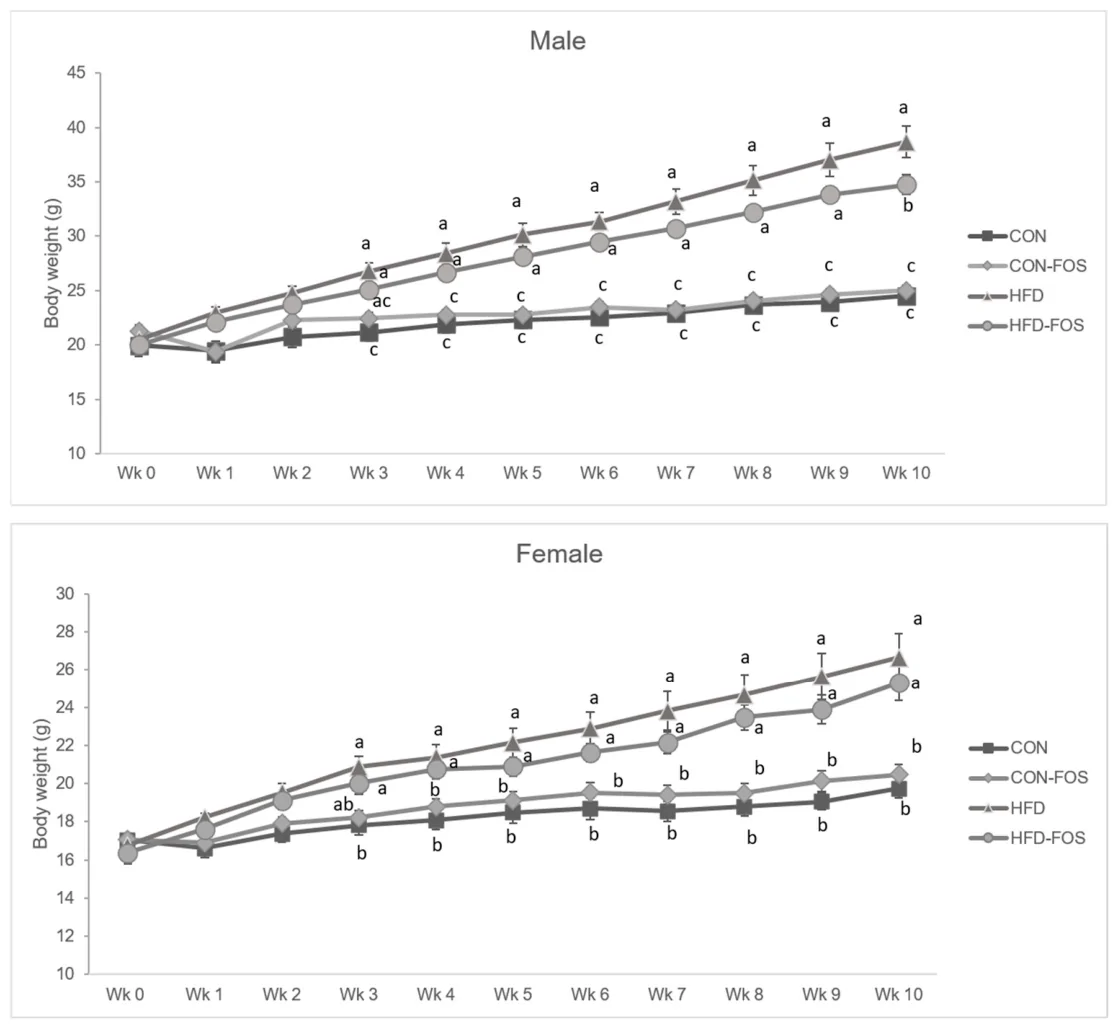
Body weight over 10 weeks of dietary intervention in male and female C57BL/6J mice. Body weight was measured weekly in mice fed control diet (CON), CON supplemented with 5% FOS (CON-FOS), high-fat diet (HFD), or HFD supplemented with 5% FOS (HFD-FOS). Data are mean ± SEM (*n* = 9–10/group/sex). Within each sex, different superscript letters (a, b, c) at a given time point indicate significant differences among diet groups (*p* < 0.05).

The body weight between CON and HFD started to deviate at week 3 of dietary intervention in both male and female. By the end of the 10-week dietary intervention, male mice fed a HFD exhibited higher average body weight (38.66 g ± 1.4 g) compared to CON mice (24.49 g ± 0.7 g), representing a 56% increase (*p* < 0.001) (Figure 1). Supplementation with 5% FOS suppressed the body weight gain in male HFD mice by 10% at week 10 of intervention (*p* = 0.025), but not in male CON mice, suggesting the potential of FOS in suppressing body weight gain in HFD mice (Figure 1). Similarly, female mice on HFD exhibited 35% greater in body weight at week 10 of dietary intervention compared to CON mice (*p* < 0.001, Figure 1). FOS, however, did not alter the body weight of female CON nor HFD mice, respectively (Figure 1).

Regardless of sex, visceral fat and serum triglyceride levels were significantly higher in HFD mice compared to CON mice. Specifically, visceral fat mass was 3.5-fold higher in male HFD mice compared to CON male mice (*p* < 0.001), indicating substantial fat accumulation induced by the high-fat diet (Table 3). Similarly, female HFD mice showed greater visceral fat mass accumulation than CON mice corresponding to a 2.8-fold increase (*p* < 0.001; Table 3). The effect of FOS on visceral fat gain was limited to female mice on HFD, by which a 43% reduction in fat mass was observed compared to the female counterpart on HFD alone (*p* = 0.001; Table 3). Though a 17% reduction was observed in FOS-treated HFD male mice compared to HFD alone, the difference did not reach statistical significance (*p* = 0.15). Similar results with serum triglyceride levels. Male HFD mice exhibited an elevated serum triglyceride levels compared to male CON mice by 31% (*p* = 0.012; Table 3). FOS supplementation reduced serum triglyceride concentrations in male HFD mice, but not CON mice, by approximately 24% (*p* = 0.019; Table 3). In contrast to male mice, no changes were detected in female mice regardless of dietary interventions.

### 3.2. FOS Alleviated HFD-Induced Insulin Resistance in Male Mice

Overall, HFD mice exhibited a descriptive trend toward higher fasting blood glucose levels compared to CON mice. Though this main effect of diet did not reach statistical significance (*p* = 0.065; Table 3). Unlike fasting blood glucose, serum insulin demonstrated lowering trend with FOS treatment in both sexes (*p* = 0.094; Table 3). Specifically, in male HFD mice, serum insulin was 7-fold higher than in CON male mice (*p* = 0.001), and that FOS tended to lower the insulin levels by 60% (*p* = 0.055; Table 3). No significant differences in insulin were detected among female diet groups (Table 3). To measure insulin resistance, HOMA-IR was calculated. Our results demonstrated that HOMA-IR among the HFD mice was normalized by FOS, indicating that insulin resistance in HFD mice was alleviated by FOS intervention in both male and female mice by 58% and 23%, respectively (Table 3).

### 3.3. Supplementation of FOS Enhanced Circulating GLP-1 Concentrations in Male HFD Mice

**Figure 2:**
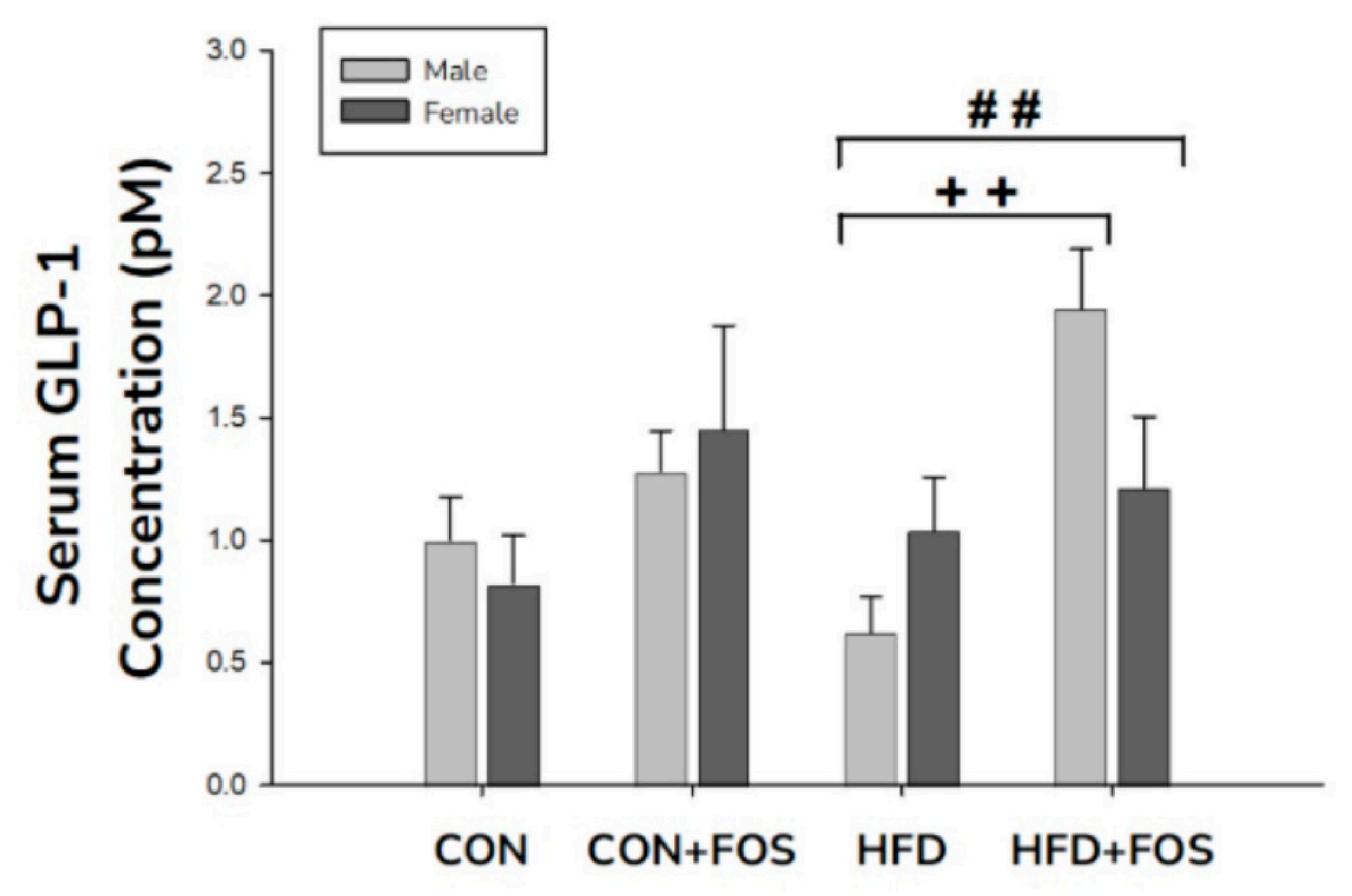
FOS increases circulating GLP-1 levels in adult C57BL/6J mice fed a HFD. Statistical differences between dietary interventions are indicated by ## *p* < 0.05. Statistical differences reflecting diet × sex interactions are indicated by ++ *p* < 0.05. Data are expressed as mean ± SEM (*n* = 5 mice per diet per sex). CON, control diet; CON + FOS, control diet with fructooligosaccharides supplementation; HFD, high-fat diet; HFD + FOS high-fat diet with fructooligosaccharides supplementation.

FOS has been reported to stimulate GLP-1 secretion from intestinal L-cells, thereby modulating insulin signaling pathways. Our data revealed that HFD alone did not alter the circulating level of GLP-1 in mice, compared to CON-fed mice. However, supplementation of FOS increased the circulating GLP-1 concentration in HFD mice, but not in CON mice (Figure 2). Specifically in male mice, serum GLP-1 level was 197.7% higher in HFD mice supplemented with FOS (HFD + FOS) compared to those on HFD alone (*p* < 0.011). In contrast, FOS did not affect serum GLP-1 level among female mice on HFD diet. This may indicate that secretion of GLP-1 by FOS intake was only stimulated under obesogenic conditions and this effect is much prevalent among male mice than female. In parallel with the results from fasting serum insulin (Table 3) where FOS-treated HFD mice demonstrated enhanced insulin sensitivity, our results may suggest that FOS may improve insulin homeostasis possibly through enhanced GLP-1 secretion.

### 3.4. FOS Modulates Colonic PI3K/Akt Signaling in a Sex and Diet-Dependent Manner

**Figure 3:**
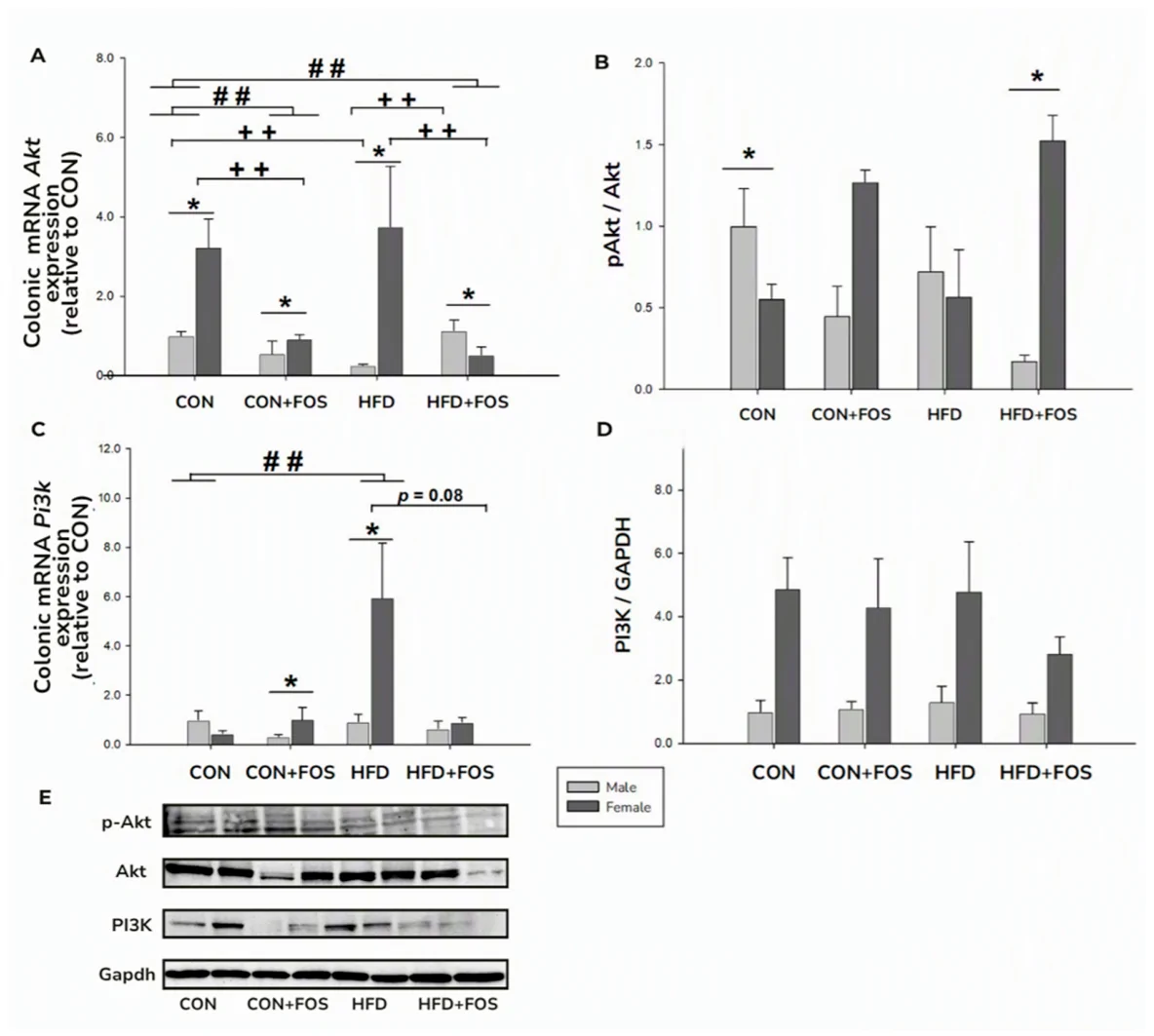
FOS differentially modulates colonic PI3K/Akt signaling in adult C57BL/6J mice. (**A**) Colonic *Akt* mRNA expression (qPCR, normalized to CON male). (**B**) Akt phosphorylation (pAkt/Akt ratio; Western blot, fold change relative to CON). (**C**) Colonic *Pi3k* mRNA expression (qPCR, normalized to CON male). (**D**) PI3K protein expression (PI3K/GAPDH ratio; (**E**) Represented Western blot images of pAkt, Akt, PI3K and Gapdh. Two-way ANOVA followed by Tukey’s post hoc test was used to assess effects of diet and sex. Statistical differences between sexes within a diet are indicated by * *p* < 0.05 Statistical differences between dietary interventions are indicated by ## *p* < 0.05. Statistical differences reflecting diet × sex interactions are indicated by ++ *p* < 0.05. Data are expressed as mean ± SEM (*n* = 4–5 mice per diet per sex). CON, control diet; CON + FOS, control diet with fructooligosaccharide supplementation; HFD, high-fat diet; HFD + FOS, high-fat diet with fructooligosaccharide supplementation. Western blot data were representative of two independent experiments. Representative immunoblots are shown for illustration; densitometric quantification and pAkt/Akt ratios were calculated from corresponding bands obtained from the same membrane for each sample.

To further determine the mechanism by which FOS-induced GLP-1 secretion, the activation of local PI3K/Akt signaling and subsequent metabolic outcomes were examined (Figure 3). Our data suggested that HFD diet did not alter colonic *Akt* mRNA expression in mice when compared to CON diet (Figure 3A). However, a robust sex effect was observed, with female mice exhibiting greater colonic *Akt* mRNA expression than male mice across all treatments (*p* < 0.001), suggesting a regulation of Akt expression in a sex-dependent manner. Specifically in male mice, HFD downregulated colonic *Akt* mRNA expression by 75% compared to CON (*p* = 0.033). Interestingly, this HFD-induced suppression was prevented by FOS supplementation with the HFD + FOS males exhibited 4.5-fold increase *Akt* mRNA expression than HFD males (*p* = 0.020), remaining comparable to CON and CON + FOS groups. In female mice, colonic *Akt* mRNA expression did not differ between HFD and CON. However, supplementation with FOS downregulated the mRNA expression of colonic *Akt* on both HFD and CON diet background, with both CON + FOS and HFD + FOS females showing 71% and 86% lower than their respective controls (*p* = 0.039; Figure 3A). At the translational level (Figure 3B), two-way ANOVA did not detect a significant main effect of diet or FOS on the pAkt/Akt ratio when sexes were collapsed. A main effect of sex approached statistical significance, showing a descriptive trend toward CON males having a higher pAkt/Akt ratio than CON females (*p* = 0.051). In addition, within females, FOS supplementation to HFD mice enhanced Akt phosphorylation, with HFD-FOS females exhibiting a roughly 2.2-fold higher pAkt/Akt ratio than HFD females (*p* = 0.021). This suggests that, although global diet effects on Akt phosphorylation were modest, FOS can selectively increase colonic Akt activation in HFD-fed females.

Unlike Akt, colonic *Pi3k* mRNA expression was influenced by both diet and sex (Figure 3C). The mRNA expression of colonic *Pi3k* in female mice was greater than males across treatments (*p* = 0.01). Among female mice, colonic *Pi3k* mRNA expression in HFD mice was upregulated by almost 6-fold compared to CON mice (*p* = 0.002), and that administration of FOS to HFD mice (HFD + FOS) exerted a lowering trend by 5-fold compared to HFD alone (*p* = 0.08), which is comparable to CON mice. No differences in *Pi3k* mRNA expression were observed in male mice (Figure 3C). At the protein level (Figure 3D), colonic PI3K abundance demonstrated a strong effect on sex, with females consistently exhibiting greater protein level of PI3K than males across diets (*p* < 0.001), while no effects of diet or FOS supplementation were detected in either sex. This dissociation between *Pi3k* mRNA expression and protein abundance may suggest post-transcriptional regulation that maintains relatively stable PI3K protein levels despite diet-induced changes in transcript abundance. Together, these data demonstrate that FOS modulates the colonic PI3K/Akt pathway in a sex-dependent manner. This may suggest a complex post-transcriptional or signaling feedback mechanism that may influence pathway output in a sex-specific manner.

### 3.5. FOS Suppresses the Upregulation of Colonic Glp1r mRNA Expression Induced by HFD

**Figure 4:**
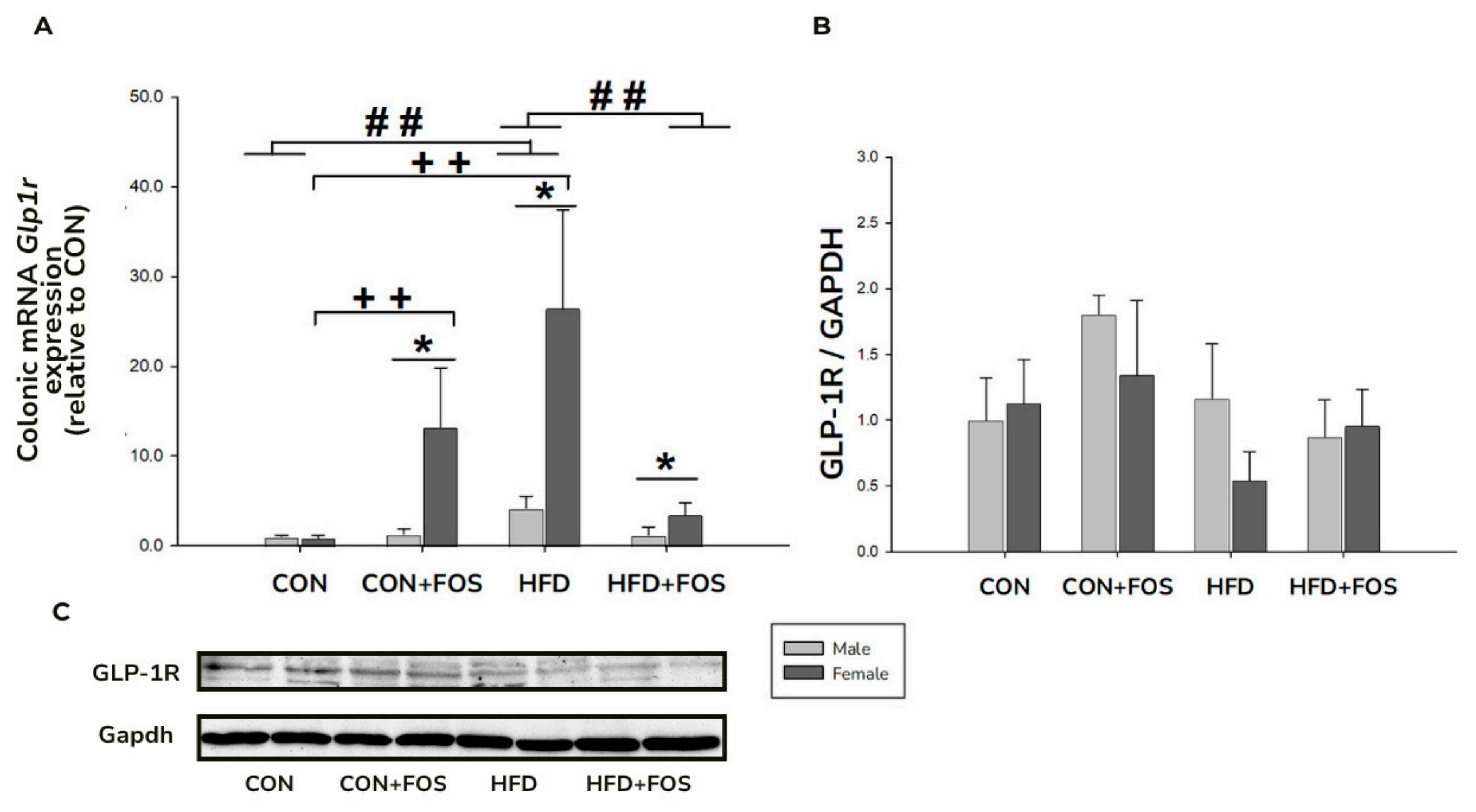
FOS attenuates the high fat diet induced upregulation of colonic *Glp1r* mRNA but not protein expression. (**A**) Colonic *Glp1r* receptor (GLP-1R) mRNA expression (qPCR, normalized to CON male). (**B**) GLP-1R protein expression (GLP-1R/GAPDH ratio; Western blot, fold change relative to CON). (**C**) Selected Western blot images of GLP-1R and Gapdh. Two-way ANOVA followed by Tukey’s post hoc test was used to assess effects of diet and sex. Statistical differences between sexes within a diet are indicated by * *p* < 0.05. Statistical differences between dietary interventions are indicated by ## *p* < 0.05. Statistical differences reflecting diet × sex interactions are indicated by ++ *p* < 0.05. Data are expressed as mean ± SEM (*n* = 4–5 mice per diet per sex). CON, control diet; CON + FOS, control diet with fructooligosaccharide supplementation; HFD, high-fat diet; HFD + FOS, high-fat diet with fructooligosaccharide supplementation. Western blot data were representative of two independent experiments. Representative immunoblots are shown for illustration; densitometric quantification were calculated from corresponding bands obtained from the same membrane for each sample.

To determine the localize effect of GLP-1 in response to FOS supplementation within the gastrointestinal tract, we further examined the mRNA and protein expressions of GLP-1R in the colon (Figure 4). Overall, sex differences were observed in which colonic *Glp1r* mRNA expression was greater in female mice than male mice (*p* = 0.001). In contrast to circulating GLP-1, colonic *Glp1r* mRNA expression in HFD mice was 30-fold higher than CON mice (*p* = 0.001; Figure 4A), and that supplementation of FOS downregulated the colonic mRNA expression of *Glp1r* of HFD mice by 7-fold (*p* = 0.006; Figure 4A), which was mainly driven by differences observed among female mice. No difference in mRNA expression was observed with *Glp1r* among male mice, indicating a phenotype change that is highly dependent on sex with female exerted stronger effect than male. In noticeable dissimilarity to the robust mRNA induction, GLP-1R protein abundance did not show significant diet or sex effects (Figure 4B).

### 3.6. FOS Suppresses the Abundance of Romboutsia ilealis in HFD Mice

**Figure 5:**
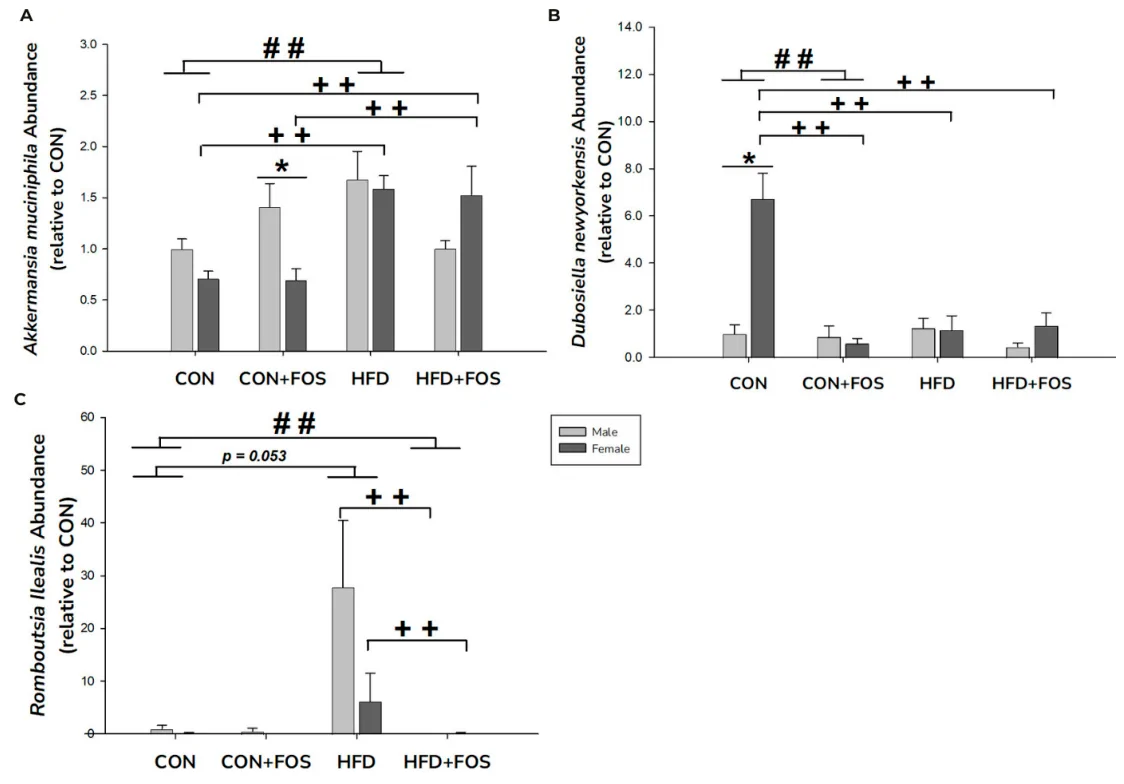
FOS modulates gut microbiota composition in a sex- and diet-dependent manner. (**A**) Cecal Akkermansia muciniphila relative abundance (qPCR, gene copies normalized to total Eubacteria, fold change relative to CON). (**B**) Cecal Dubosiella newyorkensis relative abundance. (**C**) Cecal Romboutsia ilealis relative abundance. Two-way ANOVA followed by Tukey’s post hoc test was used to assess effects of diet and sex. Statistical differences between sexes within a diet are indicated by * *p* < 0.05. Statistical differences between dietary interventions are indicated by ## *p* < 0.05. Statistical differences reflecting diet × sex interactions are indicated by ++ *p* < 0.05. Data are expressed as mean ± SEM (*n* = 5 mice per diet per sex). CON, control diet; CON + FOS, control diet with fructooligosaccharide supplementation; HFD, high-fat diet; HFD + FOS, high-fat diet with fructooligosaccharide supplementation.

To identify bacterial taxa potentially involved in FOS-associated metabolic effects, we further quantified the cecal abundance of AM, DN, RI by qPCR (Figure 5). These taxa have been linked to gut barrier function, inflammation, and metabolic regulation in previous work. In contrast to what we would expect, AM abundance was greater in HFD mice compared to CON mice regardless of sex (*p* = 0.001; Figure 5A). FOS did not alter the abundance of AM (Figure 5A). With regards to DN, the abundance was lower in FOS supplemented CON mice (*p* = 0.013) and HFD mice (*p* = 0.04), respectively, and that no difference was observed between HFD and CON mice (*p* = 0.145; Figure 5B). In line with other observation, a main sex effect was detected in which female mice exhibited greater abundance of DN than male mice (*p* = 0.038), mainly driven by the differences between CON male mice and CON female mice (*p* = 0.002). In addition, in female, but not male mice, both FOS and HFD attenuated the abundance of DN compared to CON.

We previously demonstrated that RI, a possible pathobiont, was attenuated by the combination of vitamin D and FOS. Here, our data further revealed that FOS alone is capable of suppressing the abundance of RI induced by HFD. In male mice, RI abundance was increased by approximately 28-fold in HFD compared with CON (*p* = 0.002), whereas in females the increase was about 3.5-fold (HFD vs. CON, *p* = 0.053) (Figure 5C). FOS supplementation markedly reduced RI abundance in HFD-fed mice of both sexes (overall diet effect *p* = 0.001), in line with our previous observations. Taken together, these data show that RI is strongly influenced by diet and FOS in a sex-dependent fashion, with high-fat feeding driving its expansion and FOS capable of reversing this effect, although the magnitude of change differs between males and females. The previously reported links between *Romboutsia* species, mucosal inflammation, and metabolic disturbances raise the possibility that changes in this taxon could contribute to diet- and sex-specific inflammatory and metabolic phenotype.

### 3.7. Selected Bacterial Abundance Was Associated with Host Signaling Markers

To investigate whether FOS-induced microbiota shifts are functionally linked to colonic signaling, correlation analyses were performed using pooled observations and were considered exploratory, because of the limited sample size within individual sex-by-diet subgroups. Thus, the observed associations may partly reflect between-group differences. Pearson product-moment correlation analyses were performed across all animals between bacterial abundance and colonic mRNA expression of *Glp1r*, *Pi3k*, *Akt*, and *Tlr4* (Table 4). The *Tlr4* mRNA expression data utilized for this correlation analysis was derived from our previously published cohort examining the same animals, as detailed in Brunelle et al. [37] and are included in Supplemental Table S2. As expected, RI abundance was positively correlated with *Tlr4* mRNA expression (*r* = 0.603, *p* < 0.001, *n* = 31), a crucial pro-inflammatory receptor that binds to bacterial LPS, suggesting a role for RI as a potential contributor to TLR4 activation. Interestingly, AM abundance, but not DN abundance, was also positively correlated with *Tlr4* mRNA expression (*r* = 0.39), suggesting that the expression and potentially the activation of TLR4 is driven by a collective group of bacteria, rather than a single bacteria species. Here, our data demonstrated that mRNA expression of *Glp1r* was strongly correlated with *Pi3k* (*r* = 0.935, *p* < 0.001, *n* = 36; Table 4) and *Tlr4* (*r* = 0.53) mRNA expressions, respectively. This may support our hypothesis that GLP-1R may be partially mediating the metabolic and inflammatory status observed in this study.

## 4. Discussion

The present study investigated the effects of dietary FOS supplementation on metabolic phenotypes in a high-fat diet-induced obese mouse model. The direct findings of this study are fourfold: (1) HFD-FOS improved HOMA-IR and showed a lowering trend in circulating insulin; (2) FOS selectively increased serum GLP-1 concentrations in HFD-fed male mice; (3) FOS significantly reduced the HFD-associated expansion of *Romboutsia ilealis* (RI) abundance; and (4) colonic *Glp1r*, *Pi3k*, and *Akt* markers exhibited sex-dependent transcriptional and post-translational changes. Regarding the direct metabolic phenotypes, supplementation with FOS alleviated HOMA-IR in HFD-fed mice compared to mice on HFD alone. This was accompanied by a suppression of body weight gain in HFD-fed males and a prominent attenuation of visceral fat accumulation in HFD-fed females. These sex-differentiated metabolic improvements demonstrate that FOS exerts broad beneficial effects on the obesity-associated metabolic phenotype, consistent with prior evidence that fermentable fibers improve multiple cardiometabolic indices [20,56,57]. These findings align with prior work by Nakajima et al. showing that inulin-type fructans reduce visceral adiposity and improve glucose tolerance in HFD-fed rodents [58], and with a meta-analysis by Le Bourgot et al. confirming that FOS supplementation attenuates glucose intolerance and insulin resistance [19]. The attenuation of HFD-induced visceral fat accumulation was more prominent in females but did not reach significance in males, despite the male-specific body weight suppression. Visceral adiposity is particularly relevant because it is more strongly associated with metabolic complications and inflammatory burden than subcutaneous fat depots [4,9]. These sex-differentiated improvements demonstrate that FOS exerts broad beneficial effects on the obesity-associated metabolic phenotype, consistent with prior evidence that fermentable fibers improve multiple cardiometabolic indices [59,60,61].

The selective increase in circulating GLP-1 observed in obese male mice following FOS supplementation is a central finding of this study. Dietary fibers are established promoters of GLP-1 secretion [33,34,52,62]. However, FOS increased GLP-1 only under obesogenic conditions in males, not in control-fed mice of either sex. This suggests that the microbiota to L-cell axis may be capacity-limited in lean animals [33,63]. Conversely, a HFD induces dysbiosis and compromised incretin signaling, creating a state where FOS-driven restoration of microbial metabolism can meaningfully stimulate GLP-1 production [10,32,34,64]. While this finding aligns with literature, the specific role of GLP-1 in mediating the observed insulin sensitivity improvements in our model remains inferential and requires validation with receptor antagonists. From a translational perspective, the ability of dietary prebiotics to enhance endogenous GLP-1 secretion is highly relevant given the current clinical prominence of pharmacological GLP-1 receptor agonists for obesity and type 2 diabetes management [16,62,65]. While these pharmacological therapies are highly efficacious, they can be limited by high costs, accessibility barriers, and gastrointestinal side effects [14,52]. Nutritional interventions like FOS that naturally promote endogenous GLP-1 production could therefore serve as valuable adjunct therapies, potentially complementing pharmacological interventions, or acting as accessible, population-level preventive strategies to support metabolic control.

FOS also modulated colonic GLP-1R and PI3K/Akt signaling. We observed a strong positive correlation between colonic *Glp1r* and *Pi3k* mRNA expressions (r = 0.94, *p* < 0.001). This transcriptional co-regulation suggests that colonic cells coordinate expression of the GLP-1 receptor with a core node of the PI3K/Akt signaling cascade [11,12]. At the level of PI3K/Akt, *Akt* mRNA expression was suppressed by HFD in males and restored to control levels by FOS. In contrast, HFD elevated colonic *Akt* and *Pi3k* mRNA expressions in females, while *Akt* mRNA expression remained unaltered compared to controls. Furthermore, FOS enhanced colonic Akt phosphorylation (pAkt/Akt ratio) in HFD-FOS females. Despite these robust transcriptional changes, total protein abundance of GLP-1R and PI3K remained largely stable. This mRNA and protein discordance is consistent with emerging evidence that GLP-1R is subject to substantial post-transcriptional regulation [66]. While these molecular changes are evident, positioning PI3K/Akt as a direct downstream effector pathway of this intervention remains a speculative mechanistic model based on our associative data.

Among the gut bacterial taxa examined, RI emerged as the most functionally relevant. RI was strongly expanded by HFD and significantly suppressed by FOS supplementation, consistent with prior work from our laboratory [38]. The strongest microbe and host correlation observed was between RI abundance and colonic *Tlr4* mRNA expression (r = 0.60, *p* < 0.001). TLR4 is the primary innate immune receptor for bacterial LPS, and its activation initiates the cascade that drives obesity-associated metabolic dysfunction [24,48]. More recently, RI has been shown to produce a metabolite that activates the farnesoid X receptor in L-cells to increase circulating GLP-1 [47]. While we hypothesize that FOS-mediated suppression of RI could limit inflammatory signaling via TLR4, this relationship is correlative, and direct interactions between RI and host TLR4 receptors remain to be causally demonstrated. AM abundance was unexpectedly increased by HFD in both sexes, which contrasts with most published literature [40,41,42]. However, an HFD-associated increase in AM has been reported in other models and may represent a compensatory mucosal response to metabolic stress [58,59]. In the present study, AM positively correlated with *Tlr4* mRNA but not with *Glp1r* mRNA, suggesting that AM’s primary mode of action in this model may operate through barrier reinforcement rather than direct transcriptional upregulation of the GLP-1 receptor [39].

Across all outcomes, sex emerged as an important modifier. Females exhibited higher colonic PI3K and Akt transcript levels at baseline and more prominent FOS-driven changes in Akt phosphorylation, while males showed the most robust FOS-induced increase in circulating GLP-1. These findings are consistent with evidence that male and female mice differ in diet-induced obesity susceptibility, gut microbiota composition, and SCFA profiles, in part due to sex-hormone effects on microbial ecology and host metabolism [8,36,59,61]. The metabolic efficacy of prebiotic strategies is therefore likely to differ between sexes and across metabolic states, arguing strongly for the intentional inclusion of sex as a biological variable in future prebiotic trials.

This study has important strengths and limitations. The factorial design simultaneously evaluated diet and FOS in both sexes, enabling detection of sex- and diet-dependent effects across metabolic, endocrine, molecular, and microbiota outcomes. The combined use of circulating hormone measurements, colonic qPCR, Western blotting, and targeted microbial qPCR provides a multi-layered view of how FOS influences the gut–hormone–metabolic axis. Limitations include the modest sample size (*n* = 5 per diet per sex for molecular assays), single time point, single mouse strain, and single FOS dose, which may limit generalizability and statistical power for smaller effects. Metabolically, relying primarily on HOMA-IR as a surrogate index for insulin resistance rather than utilizing dynamic physiological tests (e.g., glucose or insulin tolerance tests), as well as the lack of a broader lipid and metabolic biomarker profile, limits our comprehensive metabolic characterization. Furthermore, our microbial analysis was restricted to three targeted bacterial taxa rather than comprehensive 16S rRNA community profiling, and pooling sexes and diet groups for our correlation matrix may have masked distinct, subgroup-specific relationships. Importantly, the absence of gain-of-function or loss-of-function experiments (such as GLP-1R antagonists or PI3K/Akt inhibitors) and direct SCFA measurements constrain mechanistic inference. Therefore, our findings demonstrate correlative associations rather than a direct, causally validated relationship, and the proposed pathway remains an inferential model. Future studies incorporating SCFA profiling, GPR41/43 expression, GLP-1R antagonism, and sex-hormone manipulation will be essential to establish causality and validate the mechanistic model proposed here.

## 5. Conclusions

In conclusion, FOS supplementation improved insulin resistance and attenuated HFD-induced metabolic dysfunction in association with increased GLP-1 in obese male mice, as well as modulation of the pathobiont *Romboutsia ilealis*. A sex-dependent modulation of colonic PI3K/Akt and GLP-1R signaling was further observed, which may partially relate to the beneficial metabolic effects of FOS in our current model. Taken together, these associative outcomes suggest a potential link between FOS supplementation and a microbiota–GLP-1–PI3K/Akt axis that operates in an obesogenic- and sex-dependent manner. Human studies stratified by sex, metabolic status, and microbiota composition, incorporating direct SCFA and GLP-1 measurements, will be necessary to determine whether this gut–hormone–metabolic axis is conserved and clinically relevant for obesity management.

## Figures and Tables

**Table 1:** List of primers set used in RT-PCR for mechanistic analysis.

Target Gene	Gene Symbol /Target	Primer Sequence (5′–3′)	Reference/ Validation
*Pi3k*	*Pi3kca*	*F: CTC TCC TGT GCT GGC TAC TGT* *R: GCT CTC GGT TGA TTC CAA AAC T*	[51]
*Akt*	*Akt1*	*F: ATC CCC TCA ACA ACT TCT AGT* *R: CTT CCG TCC ACT CTT CTC TTT C*	[51]
*Glp1r*	*Glp1r*	*F: GGG TCT CTG GCT ACA TAA GGA CAA C* *R: AAG GAT GGC TGA AGC GAT GAC*	[52]
*Tlr4*	*Tlr4*	*F: GCTGGTTTACACGTCCATCG* *R: ACACCTGCCAGAGACATTGC*	[37]
*Gapdh*	*Gapdh*	*F: GGA GAA ACC TGC CAA GTA* *R: AAG AGT GGG AGA TCC TGT TG*	[37,38]

**Table 2:** List of primers set examined used in qPCR for bacterial analysis.

Primer	Primer Sequence (5′–3′)	Application	Reference/ Validation
*Akkermansia muciniphila*	*F: CAG CAC GTG AAG GTG GGG AC* *R: CCT TGC GGT TGG CTT CAG AT*	Species-specific qPCR	[53]
*Dubosiella newyorkensis*	*F: CGA GGA AGG TCT TCG GAT CG* *R: AGG ACT CAC TGC GTT GAC TG*	Species-specific qPCR	[38]
*Romboutsia ilealis*	*F: GGG GCT AGC GTT ATT CCG AA* *R: CAC CTG TCA CTT CTG TCC CCA*	Species-specific qPCR	[54]
*Universal Eubacteria*	*F: ACT CCT ACG GGA GGC AGC AGT* *R: ATT ACC GCG GCT GCT GGT*	Total bacterial DNA normalization	[55]

**Table 3:** Metabolic phenotype in male and female C57BL/6J mice after 10 weeks of dietary intervention.

Parameter	CON	CON + FOS	HFD	HFD + FOS
**Males**				
Visceral fat (% body weight)	1.81 ± 0.17 ^b^	1.69 ± 0.18 ^b^	6.37 ± 0.3 ^a^	5.29 ± 0.51 ^a^
Serum TG (mg/dL)	78.82 ± 7.42 ^b^	72.28 ± 4.72 ^b^	104.81 ± 5.51 ^a^	78.62 ± 6.93 ^b^
Blood glucose (mmol/L)	8.64 ± 1.45	11.98 ± 1.31	12.21 ± 1.06	12.94 ± 1.84
Serum Insulin ^¥^ (ng/mL)	0.18 ± 0.05 ^b^	0.22 ± 0.06 ^b^	0.71 ± 0.11 ^a^	0.29 ± 0.07 ^ab^
HOMA-IR	1.92 ± 0.50 ^b^	2.92 ± 0.58 ^b^	11.64 ± 2.24 ^a^	3.56 ± 0.94 ^b^
**Females**				
Visceral fat (% body weight)	1.65 ± 0.39 ^c^	1.52 ± 0.29 ^c^	4.62± 0.53 ^a^	2.63 ± 0.31 ^b^
Serum TG (mg/dL)	61.16 ± 3.81	65.03 ± 3.78	76.60 ± 9.05	67.63 ± 3.52
Blood glucose (mmol/L)	9.35 ± 1.77	12.20 ± 1.66	10.01 ± 0.52	14.44 ± 2.53
Serum Insulin ^¥^ (ng/mL)	0.22 ± 0.04	0.30 ± 0.06	0.54 ± 0.19	0.33 ± 0.13
HOMA-IR	2.64 ± 0.63 ^b^	3.59 ± 0.93 ^b^	7.99 ± 2.70 ^a^	4.66 ± 1.84 ^ab^

Values are mean ± SEM. ^¥^ Data were log_10_-transformed prior to analysis. Within each sex, means not sharing a common superscript letter (a, b, c) differ significantly (*p* < 0.05); groups sharing at least one letter are not significantly different. Superscript letters are assigned independently within males and females. Mice (*n* = 9–10/group/sex) were randomly assigned to either control diet (CON), CON supplemented with 5% FOS (FOS-C), high-fat diet (HFD), or HFD with 5% FOS (FOS-H) throughout the experimental.

**Table 4:** Correlation coefficients among colonic signaling markers, inflammatory mediator, gut bacterial taxa, and serum metabolic markers.

***Gene***	*PI3K*	*GLP1R*	*TLR4*	*AM*	*DN*	*RI*	*Akt*
*Pi3k*	-						
*Glp1r*	0.94 *** n = 36	-					
*Tlr4*	0.40 * n = 38	0.53 ** n = 35	-				
*AM*	0.12 *n* = 39	0.18 *n* = 36	0.35 * n = 41	-			
*DN*	−0.05 *n* = 38	−0.01 *n* = 33	−0.04 *n* = 38	−0.003 *n* = 42	-		
*RI*	0.35 † *n* = 28	0.39 † *n* = 26	0.60 *** n = 31	0.39 * n = 34	0.19 *n* = 31	-	
*Akt*	0.22 *n* = 35	−0.001 *n* = 32	−0.28 *n* = 38	−0.31 † *n* = 39	0.12 *n* = 37	−0.19 *n* = 29	-

*** *p* < 0.001; ** *p* < 0.01; * *p* < 0.05; † 0.05 ≤ *p* < 0.10 (trend). Sample sizes range from *n* = 26 to *n* = 42 depending on assay availability. Bacterial abundance = qPCR fold-change relative to total Eubacteria. Target gene expression = relative expression values calculated via 2^−ΔΔCt^ from qRT-PCR. *AM* = *Akkermansia muciniphila*; *DN* = *Dubosiella newyorkensis*; *RI* = *Romboutsia ilealis*. Note: Colonic *Tlr4* mRNA expression data were obtained from previously published work utilizing the same experimental cohort [37].

## Data Availability

All data generated or analyzed during this study are indicated in this article. The datasets generated and/or analyzed during the current study are available from the corresponding author upon reasonable request.

## Supplementary Materials

The following supporting information can be downloaded at: https://www.mdpi.com/article/10.3390/nu18193258/s1, Table S1: Composition of experimental diets (g/kg diet); Figure S1: Energy intake (kcal) over 10 weeks of dietary intervention in male and female C57BL/6J mice. Table S2. Colonic *Tlr4* mRNA expression in male and female C57BL/6 mice.

## Abbreviations

The following abbreviations are used in this manuscript:

GLP-1	Glucagon-Like-Peptide 1
PI3K	Phosphoinositide 3-kinase
Akt	Protein kinase B
FOS	Fructooligosaccharides
HFD	High-Fat Diet
SCFA	Short-chain Fatty Acids
HOMA-IR	Homeostatic Model Assessment for Insulin Resistance
AM	*Akkermansia muciniphila*
DN	*Dubosiella newyorkensis*
RI	*Romboutsia ilealis*
qPCR	Quantitative Polymerase Chain Reaction
qRT-PCR	Quantitative Reverse Transcription Polymerase Chain Reaction
ANOVA	Analysis of Variance
