# Supplementary material for: Dietary Fructooligosaccharide Is Associated with Improved Insulin Resistance, Reduced Romboutsia ilealis Abundance, and Modulation of GLP-1 Production in High-Fat Diet-Induced Obese Mice
Source: Nutrients. 2026 Oct 2;18(19):3258. doi: 10.3390/nu18193258 (PMC13666743; doi:10.3390/nu18193258)
Supplement: Supplementary file 1 [file nutrients-18-03258-s001.zip › nutrients-4481075-supplementary.pdf]

Supplementary Table S1. Composition of experimental diets (g/kg diet).

Ingredient	CON (D12450J)	CON+FOS	HFD (D12492)	HFD+FOS
Casein	200	200	200	200
L-Cystine	3	3	3	3
Corn starch	506.2	506.2	0	0
Maltodextrin 10	125	125	125	125
Sucrose	68.8	68.8	68.8	68.8
Cellulose	50	50	50	50
Orafti P95 (95% FOS)	0	55.5	0	55.5
Soybean oil	25	25	25	25
Lard	20	20	245	245
Mineral mix (S10026)	10	10	10	10
Dicalcium phosphate	13	13	13	13
Calcium carbonate	5.5	5.5	5.5	5.5
Potassium citrate, 1 H_2O	16.5	16.5	16.5	16.5
Vitamin mix (V10001)	10	10	10	10
Choline bitartrate	2	2	2	2
FD&C Yellow #5	0.04	0.05	0	0
FD&C Red #40	0	0	0	0.05
FD&C Blue #1	0.01	0	0.05	0
**Total (g/kg)**	**1055.05**	**1110.55**	**773.85**	**829.35**
**Energy (kcal/g)**	**3.8**	**3.7**	**5.2**	**4.9**
**Protein (% kcal)**	**20**	**17**	**20**	**24**
**Carbohydrate (% kcal)**	**70**	**70**	**20**	**20**
**Fat (% kcal)**	**10**	**10**	**60**	**60**
**FOS (Orafti P95, % w/w)**	**0**	**10.0**	**0**	**10.0**

Formulations based on open-source diets D12450J, D12492, and custom Orafti P95 diets provided by Research Diets, Inc. (New Brunswick, NJ, USA).

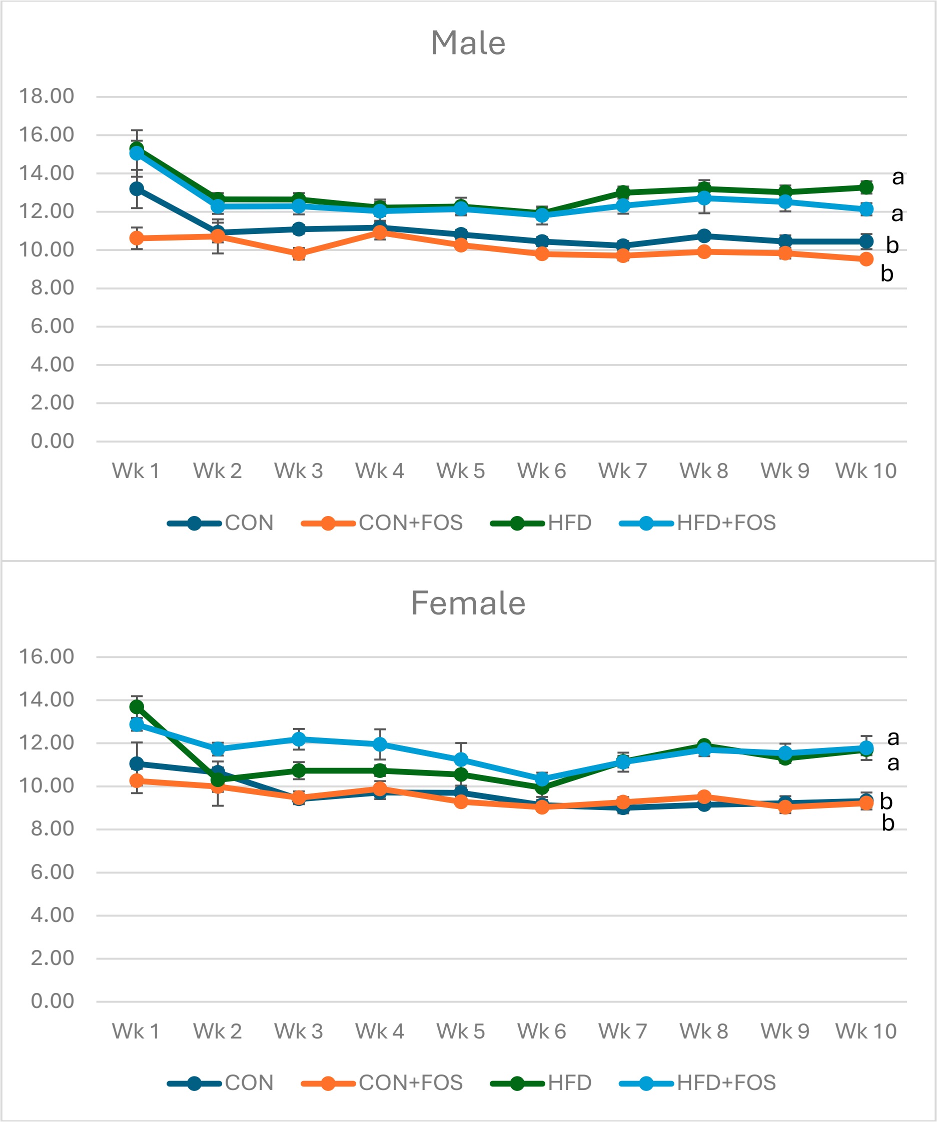

Supplemental Figure S1. Energy intake (kcal) over 10 weeks of dietary intervention in male and female C57BL/6J mice. Energy intake was measured weekly in mice fed control diet (CON), CON supplemented with 5% FOS (CON-FOS), high-fat diet (HFD), or HFD supplemented with 5% FOS (HFD-FOS). Data are mean ± SEM (n = 9–10/group/sex). Within each sex, overall significant differences ($p < 0.05$) were indicated by different letters (a, b,).

Supplemental Table S2. Colonic Tlr4 mRNA expression in male and female C57BL/6 mice.

	Male	Female
CON	1.00 ± 0.26^{a}	0.54 ± 0.09
CON + FOS	1.12 ± 0.38^{a}	1.153 ± 0.31
HFD	1.76 ± 0.32^{ab}	1.69 ± 0.11
HFD + FOS	0.63 ± 0.10^{b}	1.01 ± 0.34

Data are expressed as mean ± SEM (n = 5 mice per diet per sex). Within each sex, means not sharing a common superscript letter (a, b) differ significantly ($p < 0.05$); groups sharing at least one letter are not significantly different. CON, control diet, CON supplemented with 5% FOS (FOS-C), high-fat diet (HFD), or HFD with 5% FOS (FOS-H) throughout the experimental. **NOTE:** Part of the data is published in Brunelle et al. [37]. Data generated was used for correlational analyses as described in this study.
